# Formulation Study of a Poly(amino methacrylate) Film-Forming Solution for Transdermal Administration

**DOI:** 10.3390/pharmaceutics17010088

**Published:** 2025-01-11

**Authors:** Chiara G. M. Gennari, Antonella Casiraghi, Francesca Selmin, Francesco Cilurzo

**Affiliations:** Department of Pharmaceutical Sciences, Università degli Studi di Milano, via G: Colombo, 71, 20133 Milano, Italy; chiara.gennari@unimi.it (C.G.M.G.); antonella.casiraghi@unimi.it (A.C.); francesco.cilurzo@unimi.it (F.C.)

**Keywords:** drug delivery system, Eudragit^®^ E, film-forming solution, plasticizer, skin permeability, supersaturation, triiodothyronine, transdermal drug delivery

## Abstract

Background/Objectives: The objective of this paper is to design a novel film-forming system (FFS) based on Eudragit^®^ E PO (EuE) polymeric solutions, differing in volatile solvents (i.e., isopropanol and ethanol) and plasticizers (i.e., tributylcitrate, glycerine, triacetin and PEG 400). Methods: The physicochemical and mechanical properties of the FFS and dried films were evaluated in terms of formation time, stickiness, T_g_, tensile strength, break elongation and Young’s modulus. The in vitro skin permeation studies were conducted on formulations containing caffeine and testosterone. Results: The FFS, consisting of EuE and PEG400 in isopropyl alcohol and ethanol (80:20, *v*/*v*), exhibited rapid film formation within about 5 min and the dried film allowed a high skin permeability compared to other formulations due to the ability to increase the thermodynamic activity of both drugs. When triiodothyronine (T3) was loaded as a model of a very low soluble drug, tocopherol polyethylene glycol succinate (TPGS) was added as a co-solvent and it allowed for the improvement of T3 retention in the skin. Conclusions: Among the formulative variables, the nature and the amount of plasticizer represent the most critical variables to obtain an EuE-based film with satisfying physical and biopharmaceutical properties.

## 1. Introduction

**Figure 1:**
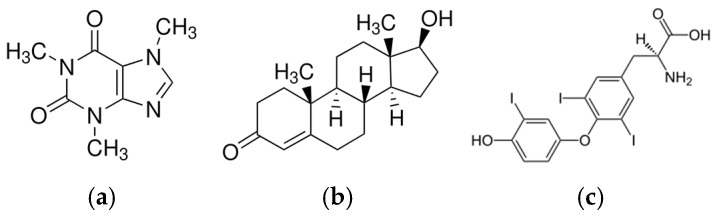
Chemical structure of (**a**) caffeine, (**b**) testosterone and (**c**) triiodothyronine.

The skin represents an attractive organ for drug delivery with several advantages, including bypassing the hepatic first-pass effect, lowering fluctuations in plasma levels and, thereby, reducing the risk of side effects or ineffective dosing. Nevertheless, its natural barrier function hinders a majority of drugs from penetrating the skin at therapeutic doses. This is the case for hydrophilic or high molecular weight substances. To overcome this issue, great efforts have been devoted to developing strategies to enhance the penetration into the stratum corneum, ranging from physical to chemical to biological approaches. One of the simplest ways to reach this goal is offered by supersaturation systems. According to Fick’s first law of diffusion, the permeation rate of a substance is directly proportional to its thermodynamic activity. In other words, the driving force is related to the increase in the degree of drug saturation in the vehicle. Among the possible approaches to obtain a supersaturated system, the rapid evaporation of a volatile solvent is the basic principle on which the design of film-forming systems (FFSs) is based. Indeed, FFSs are simple dispersions of a drug and a film-forming polymer in a skin-tolerant solvent: once sprayed onto the skin, FFSs undergo rapid metamorphosis, driven by the solvent evaporation, and the resultant polymeric matrix on the site of application would enhance the drug partition into the stratum corneum and guarantee a prolonged drug release. It appears obvious that the solvent evaporation rate plays a fundamental role; however, an overview of the literature indicates that the selection of the film-forming polymer and the plasticizer, along with the tensile properties and stickiness, are among the critical quality attributes to consider [1,2,3]. As a matter of fact, the glass transition temperature (T_g_) of the film-forming material must be adequately tuned-up. The T_g_ should be lower than the skin’s surface temperature, which is approximately 32 °C [4], to assure the formation of a uniform film with an adequate flexibility to overcome the stresses caused by the body’s movements. At the same time, the lack of stickiness should be guaranteed to avoid adhesion to cloths, and it is generally recognized that when the T_g_ is about 35–45 °C below its application temperature, a material can become sticky [4]. Undoubtedly, the choice of film-forming polymer has the greatest influence on the substantivity of the formulation. Among possible materials included in the FDA list of inactive ingredients approved to produce dosage forms to be applied on the skin [5], Eudragit^®^ E PO (EuE) is considered promising to design a FFS due to its use in transdermal patches and films [6,7], either as single film-forming component [1,8] or in combination with polyvinyl alcohol (PVA) [9]. Since no systematic investigations have been carried out to optimize the formulation development, this study aims contribute to the rational design of EuE-based FFSs, which can provide sustained and effective topical therapy. The effect of several volatile solvents (i.e., isopropanol and ethanol) and plasticizers (i.e., tributyl citrate, glycerin, triacetin and polyethylene glycol 400) were investigated. Variables including the polymer/plasticizer ratio, polymer concentration and solvent evaporation rate were evaluated with a special focus on the tackiness, elasticity and mechanical properties of the film [4]. FFSs showing no stickiness and optimal elasticity were initially loaded with the two reference drugs indicated by the OECD guideline on skin absorption, namely caffeine and testosterone (Figure 1). Then, since FFSs can be potentially also used in the treatment of skin diseases, attention was focused on the ability of such a formulation to improve the skin’s retention of drugs. This aspect, which is not yet considered in the literature, was studied by using triiodothyronine (T3, Figure 1).

Thyroid hormones (T3 and T4) are usually administered orally to treat hypothyroidism; however, they can offer multiple benefits even when administered locally, since they can exert various beneficial effects in the treatment of wounds and scars [10]. Indeed, thyroid hormones (T3 and T4) regulate the proliferation, differentiation and homeostasis of epidermal cells and influence the function of dermal fibroblasts; they are also involved in sebum production and hair growth. Nowadays, it is recognized that the “thyroid-skin connection” [11], both in the skin cells and in the hair follicles, specific receptors, co-activators and co-repressors, transporters and metabolic enzymes, are involved in the functions of hormones themselves.

Most T4 and T3 molecules circulate in the blood tightly bound to carrier proteins—thyroxine (T4)-binding globulin or transthyretin—and act as a circulating reservoir of the thyroid hormone (TH), which is not directly available for cellular uptake. Only the much less abundant unbound (free) T4 and T3 molecules enter the dermis, and the tissue concentration depends on the conversion of T4 into T3 by the deiodinases present in the keratinocytes or fibroblasts. When T3 is administered topically, it reaches the epidermis directly, avoiding increases in plasma concentration, which could cause thyrotoxicosis. Studies carried out on rodents, in which systemic thyrotoxicosis was induced, revealed an increase in the loss of both hair and collagen and a thinning of the epidermis; in fact, excess T3 levels induce the release of antiproliferative factors by fibroblasts, which act on the precursors of keratinocytes present in the basal layer of the epidermis [12]. Therefore, T3 application through topical formulations could represent a potential treatment for chronic wounds or other conditions, such as alopecia, xerosis and keratinization disorders.

## 2. Materials and Methods

### 2.1. Materials

Eudragit^®^ E PO (poly (butyl methacrylate-co-(2-dimethyl aminoethyl) methacrylate-co-methyl methacrylate, EuE); molar proportion of the monomer units 1:2:1; weight average molar mass 150 kDa) was kindly supplied by Rofarma Italia (Gaggiano, taly). Polyethylene glycol 400 (PEG), triacetin (TRI), tributhyl citrate (TBC), glycerin (GLY) and vitamin E polyethylene glycol succinate (TPGS) were purchased from Caesar & Loretz (Hilden, Germany), Sigma-Aldrich (Darmstadt, Germany), Morflex (Greensboro, NC, USA), VWR International s.r.l. (Milan, Italy) and BASF (Cesano Maderno, Italy), respectively. Testosterone (TES) and triiodothyronine sodium (T3) were purchased from Sigma-Aldrich (Darmstadt, Germany) and caffeine (CAF) was purchased from A.C.E.F. (Fiorenzuola D’Arda, Italy). All solvents were of analytical grade, unless specified.

### 2.2. Preparation of FFS

The FFSs were prepared by gradually incorporating 20% of EuE in different mixtures of isopropanol (IPA) and ethanol (EtOH) at the ratio of 90:10 and 80:20%, *v*/*v*. Increasing aliquots of plasticizers, selected among PEG, TRI, TBC, TPGS or GLY (i.e., 1, 2, 4 or 6%, *w*/*w*), were added, as summarized in Table 1. Each solution was stirred overnight to ensure the complete entanglement of the polymeric chains.

In the case of the drug-loaded FFS, CAF or TES were dissolved in the solvent mixture at a concentration of 0.2 and 5%, *w*/*w*, respectively, before adding EuE (Table 2).

In the case of formulations containing T3, the drug was added in different amounts (0.02, 0.03, 0.04%, *w*/*w*) before incorporating the polymer. To guarantee complete dissolution, 4 h passed from the moment of the addition of T3 to the following incorporation of the polymer.

### 2.3. Differential Scanning Calorimetry

To rationalize the plasticizer selection, both in terms of type and concentration, the glass transition temperature (T_g_) of dried placebo films was measured by differential scanning calorimetry (DSC, DSC1 Instruments, Mettler-Toledo, Greifensee, Switzerland, CH). About 20 mg (±0.01 mg) of preformed film was exactly weighed, sealed in an aluminum DSC pan and heated in an inert atmosphere (70 mL/min of nitrogen). Samples were scanned from 25 to 80 °C with a heating rate of 20 K/min, in order to erase the thermal history of the polymer, then cooled down to −50 °C at 20 K/min and finally re-heated to 80 °C at 20 K/min. The T_g_ was extrapolated by the inflection point’s first derivative during the second heating ramp. A pan containing aluminum oxide was used as a reference to stabilize the baseline. Instrument calibration was performed using an indium standard.

### 2.4. Mechanical Testing on Films

*Film preparation*: Placebo films were obtained by a solvent evaporation technique by using an air-forced circulation oven (Mathis LabCoater LTE-S(M), Mathis, CH), equipped with a blade coater for casting the film-forming solutions. The FFSs were spread onto a plastic liner and heated for 20 min at 32 ± 1 °C. The spreading thickness was set in order to obtain dried films of about 50 μm.

*Probe tack test*: For the evaluation of the adhesive properties, a dynamometer equipped with a 50 N load cell transducer (Instron 5965, ITW Test and Measurements Italia S.r.l., Pianezza, Italy) and a flat stainless-steel punch (6 mm diameter) were used. The probe tack test, conducted according to an internal standard procedure [13], measures the strength necessary for separating the test punch tip from the film sample surface. Briefly, the punch was placed about 0.05 mm above the film sample. Then, the punch tip was put in contact with the sample surface and a constant strength of 0.05 N was applied onto the specimens for 5 s. Finally, the punch was removed at a separation rate of 0.1 mm/s. Stress (*σ*) values were calculated according to the equation below:

(1) σ=F/A

where the variables are defined as follows:

*F* = the strength recorded during separation;

*A* = the punch surface area.

The results are expressed as the mean ± SD of four measurements for each formulation.

*Tensile tests*: A dynamometer equipped with a 50 N load cell transducer and two pneumatic jaws was used. The two jaws were placed at a distance of 7 mm and film samples (20 × 30 mm) were positioned between them. During the test, the lower jaw remains fixed in the start position, while the upper jaw, connected with the load cell, rises at a constant rate of 2 mm/min. The Young’s modulus (*Y*) was calculated as the slope of the linear portion of the strength–elongation plot. The *Y* was expressed as the force per unit area (MPa), based on four performances for each formulation.

### 2.5. In Vitro Permeation Experiments

Fresh porcine ears were obtained from a local abattoir (Lodi, Italy). The ears were removed prior to the steam cleaning process in order to preserve the integrity of the skin. Hair was gently removed with a blade, then the pig ear skin was separated from the underlying cartilage, cut into squares of about 3.5 cm^2^, sealed in evacuated plastic bags and frozen at −20 °C. Before the experiments, the samples were allowed to equilibrate at room temperature for 1 h [14].

The permeation experiments were performed using modified Franz diffusion cells (PermeGear, Hellertown, PA, USA), under non-occlusive conditions. They have a diffusion area of 0.636 cm^2^ and a receptor compartment volume of approximately 3.0 mL. In the case of formulations loaded with CAF, the receptor compartment was filled with a degassed physiological solution, and NaN_3_ (0.01%, *w*/*v*) was added as a preservative agent. For formulations loaded with TES, a mixture of the physiological solution and PEG 400 (ratio: 60:40, *v*/*v*) was used as the receptor phase. For T3, the receiving phase used for each experiment was pH = 7.4 PBS/DMSO in a 90:10, *v*/*v,* ratio.

The pig skin sample was mounted on the Franz diffusion cell, preventing the formation of air bubbles between the receptor phase and the skin. The upper and lower part of the vertical diffusion cells were sealed with Parafilm^®^ and fixed together by means of a clamp. Aliquots of FFSs were applied onto the skin surface as the donor phase, with the help of a micropipette. The receptor phase was maintained under constant stirring with a magnetic rod (1500 rpm) and the whole system was kept at 32 ± 1 °C by means of a circulating water bath. The experiments (three replicates per formulation) were performed during a 24 h period in the case of CAF and T3 formulations, and during a period of 48 h for TES ones. Aliquots of 200 μL were withdrawn at predetermined times and replaced by aliquots of the fresh receptor medium. Sink conditions were maintained during the entire experiment.

The pig-ear skin integrity was determined by electrical resistance before each experiment using a cut-off of 10 kΩcm^2^ [15].

The cumulative amount of permeated drug per unit of area was determined from the concentration of each compound in the receptor phase and plotted as a function of time. The average flux (*J*) was calculated as the slope of the permeation profile, considering the linear portion.

### 2.6. Extraction of T3 from the Membranes

As far as the T3 formulations are concerned, once the permeation experiment was completed, the Franz cells were carefully disassembled, removing the layers of Teflon and Parafilm^®^ and separating the donor compartment from the receptor one. The membrane was then swabbed and rubbed with absorbent paper soaked in IPA, in order to completely remove the overlying polymeric film, and each fragment was then washed. The excess skin was cut and removed until a circular fragment was obtained surrounding the central permeation area.

Each membrane was therefore weighed and subsequently chopped with a scalpel to obtain pieces with the smallest possible size. They were then deposited in amber glass vials, to which 2 mL of a solution was added to extract the active ingredient. The extraction solution was composed as follows: 90% water/acetonitrile/trifluoroacetic acid solution (70/30/0.1, *v*/*v*) and 10% water/methanol solution (50/50, *v*/*v*) with the addition of NaOH 400 mg/L. The samples were then heated in a water bath at 40 °C for 30 min, followed by sonication using an ultrasonic homogenizer (UP200St ultrasonic processor—Hielscher, Teltow, Germany) for 10 min, continuously, with an amplitude of 70%. After sonication, the samples were filtered with 0.45 μm PVDF membranes and the solutions were analyzed to determine the drug amount retained into the skin.

### 2.7. Drug Assay

The samples from the permeation experiments were analyzed by HPLC (HP 1100, Chemstation, Hewlett Packard, Santa Clara, CA, USA). The chromatographic conditions were as follows:

*CAF*: Column: Accucore XL^®^ C18 4 μm 4.6 × 100 mm (Superchrom S.r.l., Milan, Italy); mobile phase: water/acetonitrile/acetic acid glacial (90/10/1 *v*/*v*/*v*); wavelength: 272 nm; temperature: 25 °C.

*TES*: InertClone^TM^ 5 μm ODS(3) 4.6 × 250 mm (Phenomenex, Castel Maggiore, Italy); mobile phase: water/acetonitrile (40/60 *v*/*v*); wavelength: 241 nm; temperature: 40 °C.

*T3*: InertClone^TM^ 55 μm ODS 100 Å, 4.6 × 150 mm (Phenomenex, Castel Maggiore, Italy); mobile phase: water (TFA 0.1%)/acetonitrile (75/25 *v*/*v*); wavelength: 230 nm; temperature: 25 °C.

Injection volume: 20 μL; flow rate: 1.0 mL/min.

The retention time was approximately 2.0 min for CAF, 5.9 min for TES and 10 min for T3. The methods presented good linearity and precision in the required concentration ranges (0.01–50 μg/mL, R^2^ ≈ 1.00000 for CAF; 0.02–50 μg/mL, R^2^ ≈ 0.99999 for TES; 0.02–5 μg/mL R^2^ 0.99993 and 5–100 μg/mL R^2^ 0.99989 for T3).

## 3. Results and Discussion

### 3.1. Formulation Study

In order to obtain a film that meets the requirements for assuring a prolonged residence time on the skin, several aspects should be considered. First, the concentration of the polymer solution in volatile solvents should be considered. The preliminary screening of the placebo solution evidenced that a 20% solution of EuE in a IPA:EtOH mixture was worthy of further consideration, as it allowed for the formation of non-sticking homogeneous films within 10 min. Regarding the glass transition temperature (T_g_) of the film-forming material, it should be lower than the temperature of the skin’s surface, which is approximately 32 °C [5], to assure the formation of a uniform film with an adequate flexibility to endure stresses caused by the body’s movements. At the same time, the lack of stickiness should be guaranteed to avoid adhesion to cloths, and it is generally recognized that when the T_g_ is about 35–45 °C below its application temperature, it can become sticky [16]. Thus, the suitable T_g_ value should range from −3° to 25 °C. Since EuE has a T_g_ around 50 °C, the addition of a plasticizer was essential. Secondly, the evaporation rate of solvents should be fast enough to lead to the formation of homogeneous and transparent films for assuring both a reproducible drug absorption and patient compliance.

**Figure 2:**
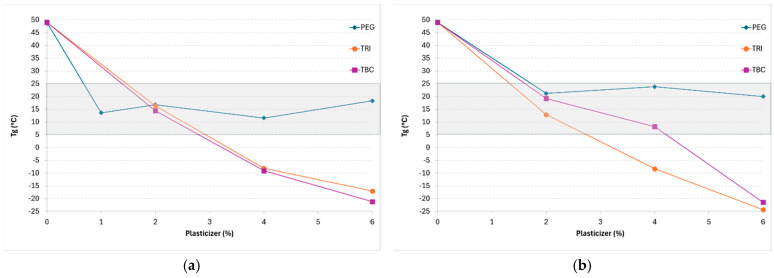
Variation in T_g_ of EuE as a function of plasticizer concentration. The FFSs were prepared by dissolving 20% EuE in IPA:EtOH at the volumetric ratio of (**a**) 90:10 and (**b**) 80:20. The grey background represents the suitable T_g_ range to assure the formation of a proper film and avoid stickiness.

Based on these considerations, the effect of different plasticizers (i.e., GLY, PEG, TRI, TBC and TPGS) at various concentrations was evaluated. Formulations containing GLY were discarded, since this plasticizer led to inhomogeneous and opaque films. In the case of TRI, TBC and TPGS, the T_g_ of the EuE films decreased when the plasticizer concentration increased, independently of the solvent composition (Figure 2a,b). TRI and TBC had a superimposable effect on the T_g_ and the 2% concentration was the optimal one; on the other hand, TPGS caused a deep depression of the T_g_ even at the lowest concentration.

**Figure 3:**
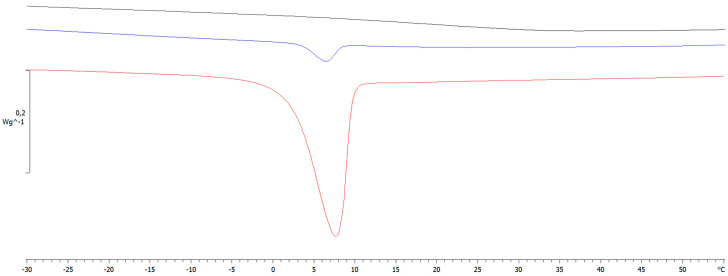
DSC traces of mixtures in IPA:EtOH at 80/20 *v*/*v* containing EuE and 1% (black line), 4% (blue line) and 6% (red line) of PEG. The segregation of PEG is evidenced as an endothermic peak at about 2–3 °C due to the melting of PEG not interacting with EuE.

PEG was not effective in reducing the T_g_ of the polymer at all concentrations tested. Moreover, at PEG concentrations higher than 2%, the DSC analysis also showed an endothermic peak at about 7 °C, which was attributed to the melting (T_m_~4–8 °C) of PEG molecules not involved in the entanglements with EuE (Figure 3).

These data suggest that PEG can act as plasticizer in a narrow range; above a critical concentration, phase separation with plasticizer exclusion occurs.

The results of film stickiness measured by the “probe tack test” are reported in Table 1. The adhesive values (σ) do not depend only on the amount of plasticizer, but also on the composition of the solvent mixture, leading to a different spatial organization of the polymeric chains upon evaporation.

As a matter of fact, formulations containing TRI or TBC at 2%, *w*/*w*, obtained starting from an IPA:EtOH 90:10, *v*/*v*, solvent mixture, led to the formation of films characterized by a higher tackiness with respect to the corresponding formulations obtained starting from an 80:20, *v*/*v*, solvent mixture (Table 1). The elastic properties of the formed films were also determined by tensile strength tests and expressed in terms of *Y* (Table 1). To adapt to the movements of the body and guarantee a prolonged contact to the site of application, EuE films should have a Young’s modulus lower than that of the human stratum corneum (*Y* = 55.4 MPa [5]). All values were at least an order of magnitude lower than the threshold value reported above, even if a slight difference related to the solvent mixture is noticeable (Table 1). In particular, the formulations composed of an IPA:EtOH 90:10, *v*/*v*, mixture resulted in more flexible films with respect to the corresponding formulations composed of an 80:20, *v*/*v*, solvent mixture (Table 1). As expected, the P8 formulation, having the lowest T_g_ values, resulted in the stickiest formulation; therefore, its *Y* modulus was not determined.

Based on the overall data, IPA:EtOH 80:20, *v*/*v*, and 2% of plasticizers were considered suitable to provide films with the desired characteristics and model drugs, i.e., CAF and TES, were loaded to study the influence of each specific plasticizer on the permeation profile through the skin.

### 3.2. In Vitro Permeability Studies

To test the performance of the FFSs containing CAF or TES, a preliminary study was carried out to determine the optimal volume of the FFS to be loaded in the donor compartment of the Franz cell. Since TES is a poorly permeable compound [17] with a relatively high solubility in the used volatile solvents, its concentration in the FFS was increased by 25 times with respect to CAF. Also, at this concentration, it was detectable and quantifiable only after 7 h and 24 h, respectively.

By comparing the skin permeation data of two drugs, one of the main pieces of evidence concerns the volume of the FFS deposited on the human skin sample and the plasticizer’s effect on the fluxes of the tested drugs (Table 2). First, independently of the drug, increasing the FFS volume from 50 to 100 μL, the amount of the permeated drug increased and at the same time the data variability decreased, probably because the higher volume would favor the spread of the solution on the skin and, consequently, the formation of a homogenous and transparent film [18]. Then, the use of PEG seemed to increase the fluxes of CAF and decreased the lag time from about 4 h to 3 h. (Table 2). Considering that this plasticizer is not a skin penetration enhancer, its effect could be attributed to a change in the thermodynamic activity of CAF loaded into the films.

To verify this hypothesis, the solubility parameter (*δ*) of each component and dried films was calculated according to Fedor’s group substitution method [19], since the drug solubility into the film was not easily quantifiable. In the case of TES, the *δ* values indicate that the thermodynamic activity appeared as the main factor governing the skin permeability, since the fluxes obtained with TES1 and TES2 formulations were higher than those obtained with the TES3 formulation, according to the *δ* values (Table 3).

On the contrary, in the case of CAF, the thermodynamic activity of the drug in the formed film cannot be considered the key factor in determining the skin permeability of CAF, since the Δδ^2^ value was the lowest for the CAF3 formulation. Thus, the improved permeability efficiency of CAF was explained by considering that the volatile solvents, before evaporating, can carry the drug in the superficial layer of the stratum corneum. As the contact time with the skin increases, the solvent and the drug would reach a greater penetration depth. In other words, it can be expected that the time required for a complete film formation is inversely proportional to the permeation rate of the drug [2]. In the present study, loading a greater amount of the donor phase increased the time required for the complete evaporation of the solvent and the contact with the skin lasted for a longer period. As a result, the flux and total amount of the active ingredient that permeated through the skin increased, as well.

This preliminary screening led to the identification of PEG as the best plasticizer for the design of T3-loaded FFSs, since it should allow the formation of films with suitable physical properties and guarantee the highest thermodynamic activity of this very liposoluble drug. Indeed, the main challenge in the formulation of T3 resulted in a very low drug solubility. Despite the presence of organic solvents and PEG, to reach the target concentration of 0.04%, *w*/*v*, the addition of a solubilizer was mandatory. TPGS is a non-ionic water-soluble derivative of vitamin E, which is found to enhance the solubility of insoluble drugs thanks to the formation of micelles (CMC = 0.2 mg/mL [20]). Additionally, some authors also report a permeation-enhancing effect of TPGS itself [21,22]. However, considering the massive plasticization effect of TPGS on EuE, the effect of TPGS on the flexibility of films made of EuE and 1% PEG obtained from an IPA:EtOH mixture in a 90:10 ratio was verified. In formulations P10 and P11, the addition of TPGS reduced the ability of PEG to plasticize the polymeric film, causing a slight increase in the T_g_ values from about 14 °C to about 20–23 °C (Table 4); the highest content of TPGS was effective as a plasticizer. Since in all formulations, the T_g_ and stickiness values were in the acceptable range, 0.04%, *w*/*v*, of T3 was loaded in P10 and P12.

Being a surface-active molecule (HLB = 14.37), TPGS favored the homogenous spread of the FFS on the skin mounted on the Franz diffusion cells not only at the volume of 50 μL, but also at 25 μL, allowing the reduction in the volume to spread on the skin with respect to other formulations without the surfactant.

**Figure 4:**
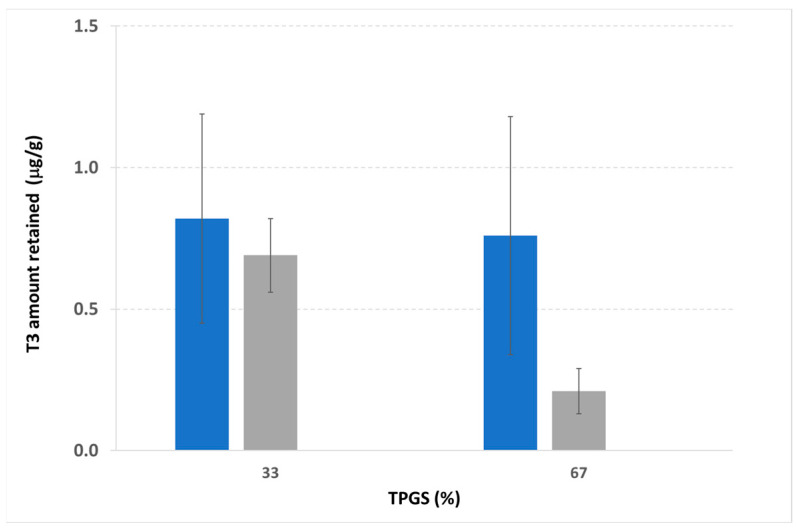
T3 amount retained in the pig skin 24 h after the application of FFS in IPA:EtOH at the 90:10 ratio. Three sets of experiments were carried out for loading on the donor compartment of the Franz diffusion cell 25 μL (blue bars) or 50 μL (grey bars) of FFS. Data are expressed as a function of the TGPS relative ratio with PEG.

In all cases, the amount of T3 detected in the receiving compartment of the Franz cells was below the limit of detection; therefore, none of the tested formulations could allow for drug permeation through the skin. Figure 4 shows the results obtained following the extraction of the active ingredient from the pig skin samples.

The application of 25 μL on the skin, independently of the PEG:TPGS ratio, allowed for an improved T3 retention in the skin. It can be assumed that the lowest volume resulted in the fastest evaporation rate. In other words, this condition would favor the formation of a supersaturated system. Increasing the volumes, the skin retention of T3 into the skin after 24 h was dependent on the TPGS:PEG ratio. This feature can be attributed to the increase in the flexibility of the EuE chains, induced by the combination of TPGS and PEG (Table 4), which can facilitate the contact between the film and the skin and, consequently, the drug distribution into the skin.

## 4. Conclusions

In the design of FFSs, the nature and the amount of plasticizer represent the most critical variables for the elasticity and stickiness of the film after solvent evaporation. These properties can not only influence the formation of a supersaturated system, but also the acceptability by the patient. In this study, PEG was found to be the most suitable plasticizer for EuE dispersed in a mixture of IPA and EtOH. Whenever the drug has a limited or poor solubility, the formulation can also comprise a surfactant, which can also act as a permeation enhancer. This strategy was exploited for T3, in which the combination of TPGS and PEG allowed for an improvement in the biopharmaceutical performances of FFS, especially in terms of solubility, spreadability and accumulation into the skin. The results obtained using the optimized FFS containing T3 at the concentration of 0.04%, *w*/*v*, were superimposable to those obtained by using polymeric nanofibers for the topical and prolonged release of T3 with the typical advantages of a solution [10]. Furthermore, as T3 does not permeate through the skin after 24 h, potential issues related to thyrotoxicosis can be excluded.

## Figures and Tables

**Table 1:** Adhesive values (σ) and Young’s modulus (Y) of placebo films differing in terms of plasticizer type and concentration and solvent system. (n.d.: not determined).

FFS no.	IPA/EtOH Ratio	Plasticizer (%)	T_g_ (°C)	σ (KPa)	Y (MPa)
P1	90:10	PEG (1)	13.6 ± 0.1	4.45 ± 1.24	2.10 ± 0.80
P2		PEG (2)	16.8 ± 0.7	0.05 ± 0.01	1.00 ± 0.23
P3		TRI (2)	16.3 ± 1.0	14.47 ± 2.58	0.68 ± 0.18
P4		TBC (2)	14.4 ± 1.3	21.17 ± 0.70	0.68 ± 0.37
P5	80:20	PEG (2)	21.2 ± 0.1	1.98 ± 0.43	2.05 ± 0.71
P6		TRI (2)	12.9 ± 2.2	1.42 ± 0.58	1.47 ± 0.62
P7		TBC (2)	19.2 ± 1.3	0.28 ± 0.03	6.91 ± 2.68
P8		TBC (4)	8.2 ± 2.7	66.61 ± 1.13	n.d.

**Table 2:** Cutaneous permeation data of caffeine (CAF) and testosterone (TES) obtained using different volumes of FFS made of 20% EuE as donor solution. The values are expressed as mean ± standard deviation (*n* = 3).

Form.	Drug (%)	IPA:EtOH (*v*/*v*)	Plasticizer	Donor Vol (μL)	J (μg cm^−2^ h^−1^)	Q24 (%)
CAF1	CAF (0.2)	80:20	TBC	50	0.32 ± 0.06	8.62 ± 0.81
				100	1.02 ± 0.56	18.39 ± 3.91
CAF2			TRI	50	0.52 ± 0.22	13.15 ± 5.14
				100	0.87 ± 0.17	20.15 ± 3.12
CAF3			PEG	50	0.91 ± 0.24	17.46 ± 4.17
				100	3.65 ± 1.91	50.12 ± 11.63
TES1	TES (5.0)	80:20	TBC	50	0.145 ± 0.065	0.032 ± 0.024
				100	0.310 ± 0.025	0.067 ± 0.008
TES2			TRI	50	0.182 ± 0.084	0.051 ± 0.047
				100	0.436 ± 0.075	0.113 ± 0.051
TES3			PEG	50	0.064 ± 0.041	0.006 ± 0.001
				100	0.206 ± 0.069	0.039 ± 0.019

**Table 3:** Solubility parameters of components and dried placebo films.

Component	*δ* (MPa^1/2^)		
EuE	19.2		
TBC	18.1		
TRI	20.8		
PEG	36.5		
CAF	28.9		
TES	22.6		
**Dried placebo films**	***δ* (MPa^1/2^)**	**Δδ^2^** **_CAF_**	**Δδ^2^** **_TES_**
with TBC	19.1	9.8	3.5
with TRI	19.4	9.6	3.3
with PEG	21.0	8.1	1.8

**Table 4:** Main physical and mechanical properties of FFS containing PEG and TPGS in different ratios.

Form. ID	PEG:TPGS Ratio	T_g_ (°C)	σ (KPa)	Y (MPa)
P9	100:0	13.6 ± 0.1	4.45 ± 1.24	2.1 ± 0.8
P10	67:33	23.5 ± 0.5	2.98 ± 0.51	1.3 ± 0.4
P11	50:50	20.4 ± 0.4	8.99 ± 1.97	1.8 ± 0.9
P12	33:67	7.8 ± 3.8	7.9 ± 2.6	0.7 ± 0.3

## Data Availability

Data are available upon request to the authors.
